# Cases Illustrating the Practical Use of the Ophthalmoscope, as a Means of Diagnosis, in Certain Diseases of the Eye

**Published:** 1859-03

**Authors:** E. L. Holmes

**Affiliations:** Chicago, Ill.


### ARTICLE III.

### CASES, ILLUSTRATING THE PRACTICAL USE OF THE OPHTHALMOSCOPE, AS A MEANS OF DIAGNOSIS, IN CERTAIN DISEASES OF THE EYE.

BY E. L. HOLMES, M.D., OF CHICAGO, ILL.

The introduction of the ophthalmoscope, has formed a new era in the history of ophthalmic science. Before its use had been made known, the diagnosis of many diseases of the eye was attended with as great a degree of uncertainty as that of diseases of the chest previous to the application of the principles of auscultation and percussion.

Any one, who can admire the accuracy with which the skillful physician can trace the progress of disease, by the simple means discovered by Avenbrugger and Laennec, must appreciate the beauty and utility of the invention of Helmholz, by the aid of which the physician can, with his own vision, clearly penetrate the delicate structures of the human eye, witness from day to day the morbid changes which take place in the choroid, in the retina and its vessels, in the papilla of the optic nerve, the vitreous humor and the lens.

The following cases, which have lately fallen under my observation, will serve to illustrate the practical use of the instrument.

If any of the readers of the *Journal* may not have had an opportunity to examine it, or have not read a description of its construction, I would refer them to a brief article upon the subject in the last volume of the *Journal* (March, 1858).

*Case I. Concussion of the Eye.*—A robust, healthy, young man, applied to me for advice, four months after receiving a severe blow upon left eye, stating that, although there was at the time a sudden confusion of vision, no external signs of injury were apparent, except slight ecchymosis in the adjacent tissues, which soon yielded to simple remedies, without much improvement of vision. During the past three months, patient has observed no favorable change. Every object in nearly the whole field of vision, appears (with the healthy eye closed) enveloped in a dark cloud, in which are numerous black spots, which remain fixed in the same relative position, and consequently are not to be regarded as *floating bodies* in the vitreous humor. Patient complains of no pain in the eye, except after reading or writing. At present the iris acts readily under the influence of light, and the power of adjusting the focus of the eye appears normal.

The ophthalmoscope reveals a small, circular, semi-opaque disk, with irregular edges, situated just behind the lens, and, possibly, lying in contact with its posterior convex surface. This disk, of chocolate color, except in small points, which are much darker (corresponding to the black spots observed by the patient), is undoubtedly produced by the extravasation of blood from the vessels of the anterior portion of the eye. This was not sufficiently extensive to produce disorganizing inflammation, but simply to effuse a small quantity of blood behind the lens, the serum of which became absorbed, leaving the coloring matter in a position to disturb vision as above described.

In my opinion, little benefit can be hoped from treatment.

*Case II. Retinal Amaurosis.*—A young woman, twenty-six years of age, of somewhat feeble constitution, has been affected for more than eight years, with constantly-progressing amaurosis, for which she was several months under treatment, at the New York Eye and Ear Infirmary, but without benefit. Since the commencement of the disease, patient has experienced a slight, dull pain in and around each eye, which, however, during the past year, has subsided in a great measure. She is unable to distinguish small objects, although she can do most kinds of housework. Pupils are slightly dilated, and not very sensitive to the influence of light.

An examination with the ophthalmoscope demonstrates, in the right eye (which is much the less serviceable to the patient) the existence of numerous, irregular, black patches of pigment. The outlines of the papule of the optic nerve are not so well defined as in health. The vessels of the retina in various places are more or less obscured by what appears to be lymph, deposited in the substance of the membrane by inflammatory action. The mere fact that black pigment, even in such quantities as found here, is deposited beneath the retina, is not enough to explain the loss of sight, since we often see similar appearances with little functional derangement. But this deposition is frequently accompanied with other unpleasant circumstances, such as pain in and around the eye, attended with a feeling of pressure and vertigo—symptoms of congestion.

In this case, these symptoms, accompanying the peculiar condition of the retinal vessels, plainly show that an inflammatory process has been going on in the choroid and retina.

The portions of the retina near the optic nerve are less affected than those nearer the cornea.

The left eye presents the same general aspect, although the morbid changes are somewhat less marked. The prognosis is unfavorable, and, unfortunately, scarcely any good results are to be expected from treatment.

[To be continued.]

